# Design and Characterization of Bioactive Bilayer Films: Release Kinetics of Isopropyl Palmitate

**DOI:** 10.3390/antibiotics9080443

**Published:** 2020-07-24

**Authors:** Ângelo Luís, Eugenia Gallardo, Ana Ramos, Fernanda Domingues

**Affiliations:** 1 Centro de Investigação em Ciências da Saúde (CICS-UBI), Universidade da Beira Interior, Avenida Infante D. Henrique, 6200-506 Covilhã, Portugal; egallardo@fcsaude.ubi.pt (E.G.); fdomingues@ubi.pt (F.D.); 2 Laboratório de Fármaco-Toxicologia, UBIMedical, Universidade da Beira Interior, Estrada Municipal 506, 6200-284 Covilhã, Portugal; 3 Departamento de Química, Faculdade de Ciências, Universidade da Beira Interior, Rua Marquês d’Ávila e Bolama, 6201-001 Covilhã, Portugal; ammr@ubi.pt; 4 Materiais Fibrosos e Tecnologias Ambientais (FibEnTech), Universidade da Beira Interior, Rua Marquês d’Ávila e Bolama, 6201-001 Covilhã, Portugal

**Keywords:** bilayer films, zein, pullulan, licorice essential oil, isopropyl palmitate, antioxidant activity, antibacterial properties

## Abstract

Active packaging incorporating antioxidants and antimicrobials is creating a niche in the market and becoming increasingly important. The main goal of this work was the design of bioactive bilayer films (zein/pullulan) incorporating licorice essential oil. The bilayer films were fully characterized in terms of their chemical, physical, barrier, antioxidant, and antimicrobial properties. Furthermore, the release kinetics of isopropyl palmitate, the major compound of the licorice essential oil, was evaluated by HPLC-DAD (high-performance liquid chromatography coupled to diode-array detector). Scanning Electron Microscopy (SEM) micrographs of cross-sections of the bilayer films clearly show the two layers of the films. Besides presenting the capacity to scavenge free radicals and to inhibit the lipid peroxidation, the developed bilayer films were also able to inhibit the growth of known foodborne pathogens (*Enterococcus faecalis* and *Listeria monocytogenes*). The release kinetics profile of isopropyl palmitate from bilayer films incorporating licorice essential oil demonstrated that in 50% ethanol at room temperature, the release was more effective, suggesting that the bilayer films will be more efficient if applied to package semi-fatty food.

## 1. Introduction

In response to changes in market trends and increasing consumers demand for high quality, safe and extended shelf-life of food products, active packaging is creating a niche in the market and becoming increasingly important. Active packaging is defined as a type of packaging in which the package, the product, and the environment interact to extend the shelf-life or improve safety and convenience, or sensory properties, while maintaining the quality and freshness of the packaged food [1]. Active packaging, incorporating antimicrobials (antimicrobial packaging) and antioxidants (antioxidant packaging), has become one of the most rapidly developing research areas in food technology [2].

To preserve food quality, several packaging materials are used, with plastics being the most employed. However, after the service-life, the solid waste of synthetic polymer materials creates a considerable problem for the environment. In most parts of the world, there are special rules and regulations to dispose plastic wastes and to recycle synthetic polymers. Thus, to preserve the environment and reduce the use of petroleum-derived materials, many researchers are developing biocomposites from renewable resources, such as cellulosic materials and biopolymers [3].

Research has focused on developing novel packaging strategies able to retain the active agent in the polymeric network and control its release. One of the most diffused techniques is the use of multilayer films [4], specifically bilayer films are a promising option to improve their overall properties. Using biodegradable polymers, a bilayer combination can complement the film-forming properties of each polymer, taking advantage of the best characteristics of each individual one [5,6]. Unlike blends, bilayer films present a heterogeneous structure where the intrinsic properties of each polymer are preserved on the corresponding layer of the film. Some of those bilayer films presented improvements in the mechanical, optical, and barrier properties when compared with the pure polymer films [7].

Recently, our research group developed functional films incorporating licorice (*Glycyrrhiza glabra* L.) essential oil as active agent [8,9]. Licorice is GRAS (Generally Recognized As Safe) by Food and Drug Administration in the United States (FDA), indicating that there is no evidence in the available information that demonstrates a hazard to the public when used at levels now applied and in the manner now practiced [10]. Furthermore, isopropyl palmitate, identified as the major compound present in licorice essential oil (71.8%) [8], is generally used in the food industry. FDA considers that this compound is not ecotoxic and classifies it as not expected to be potentially toxic or harmful, presenting a low human health priority [11].

Considering this context, the present work describes the design of bilayer active films composed by one layer of pullulan and another layer of zein. Zein is a class of alcohol-soluble prolamine storage proteins in maize. Based on the long history of zein usage in food, it was approved by FDA as a GRAS excipient for film coating of pharmaceuticals, mainly involving tablets [12]. Pullulan is one of the industrially important polysaccharides, which possesses potential applications in food industries. It is an edible polymer without any toxicity or carcinogenicity [13]. It is also declared safe by FDA for use in food applications and has GRAS status [13]. The licorice essential oil (active agent) was incorporated on pullulan internal layer, taking advantage of its hydrophilicity, which will facilitate the release of isopropyl palmitate (major compound) to the packaged food product. The external layer is composed by zein, whose films have good water barrier properties due to its hydrophobicity [5]. After the production of the bilayer films, they were fully characterized in terms of their chemical, physical, barrier, antioxidant, and antimicrobial properties. Furthermore, the release kinetics of isopropyl palmitate was evaluated by HPLC-DAD (high-performance liquid chromatography coupled to diode-array detector).

## 2. Materials and Methods

### 2.1. Reagents

Licorice essential oil (*Glycyrrhiza glabra* L., *Leguminosae*/*Fabaceae*) was obtained from Best Formula Industries (BF1, Kuala Lumpur, Malaysia). The essential oil (Pure Essential) was extracted from trunks of the plant by steam distillation. The purity of this essential oil was tested, and its quality ensured to be consistent with the standards of the European Pharmacopoeia, being suitable to be used in products for human consumption. The chemical composition of the licorice essential oil, analyzed by Gas Chromatography coupled to Mass Spectrometry (GC-MS; Perkin Elmer Clarus 600, Shelton, CT, USA), and its bioactivities, namely antioxidant and antibacterial, were previously reported [8]. Isopropyl palmitate (CAS Number: 142-91-6), identified as the major compound of licorice essential oil (71.8%) [8] was obtained from Sigma-Aldrich (Saint-Louis, MO, USA). Zein from maize (CAS Number: 9010-66-6), presenting a molecular weight of 22–24 kDa, was also supplied by Sigma-Aldrich (Saint Louis, MO, USA). Pullulan (CAS Number: 9057-02-7) and oleic acid (CAS Number: 112-80-1) were purchased from TCI Europe N.V. (Belgium). Glycerol (anhydrous extra pure) (CAS Number: 56-81-5) was supplied by Merck (Darmstadt, Germany). Tween 40 (polysorbate 40) (CAS Number: 9005-66-7) was supplied by Riedel-de Haën (Germany). Xanthan gum (TEXTURAS, Solé Graells SA, Barcelona, Spain) for culinary purposes was purchased on a local supermarket.

### 2.2. Preparation of Bilayer Films

#### 2.2.1. External Layer: Zein Films

The film-forming solution was prepared by dissolving 5 g of zein powder in 50 mL of 80% (*v*/*v*) ethanol (zein 10%; *w*/*v*) under magnetic stirring at 250 rpm for 5 min at room temperature [9]. Then, 1.25 g of plasticizer (25%, *w*/*w*, relatively to dry zein powder), composed by a mixture of oleic acid/glycerol (3:1) were added and stirred for 30 min at 60 °C and 300 rpm [14]. This mixture was degasified under vacuum, with the films being obtained by the casting method. For this purpose, the film-forming solution was spread on polystyrene Petri dishes (≈5.5 mL), which were placed into a ventilated oven to dry the mixture (1 h, 60 °C) [9,14].

#### 2.2.2. Internal Layer: Pullulan Films Incorporating Licorice Essential Oil

Pullulan solution (2%, *w*/*v*) was prepared by dissolving 2 g of pullulan powder in 100 mL of deionized water at room temperature and stirring with a magnetic stirrer for 5 min at 250 rpm. After that, 15% glycerol (*w*/*w*, relatively to dry pullulan powder) was added as a plasticizer to the pullulan solution and stirred for 30 min at 50 °C [15,16].

The licorice essential oil (30%, *w*/*w*, relatively to dry pullulan powder) was separately mixed with 2% Tween 40 (*w*/*w*, relatively to dry pullulan powder) used as emulsifier and 5% xanthan gum (*w*/*w*, relatively to dry pullulan powder) used to stabilize both the matrix solution and the emulsion in 10 mL of deionized water during 5 min under magnetic stirring [15,16].

Then, the mixture of licorice essential oil/Tween 40/xanthan gum was added to the pullulan solution and was further mixed at 50 °C during 10 min and 300 rpm. After that, the solution was homogenized for 4 min at 7600 rpm with a rotor/stator homogenizer (IKA T25 Digital Ultra-Turrax, Staufen, Germany), and for another 4 min at 12,000 rpm. The solution was finally degasified under vacuum and the films were obtained by casting method, spreading ≈ 12 mL on the Petri dishes containing the zein-based films previously prepared. These sets were then placed in a ventilated oven at 60 °C for 3 h [15,16]. Control films were also prepared under the same conditions but without adding the licorice essential oil.

### 2.3. Characterization of Films

#### 2.3.1. Infrared Spectra

FTIR (Fourier-Transform Infrared Spectroscopy) spectra of bilayer films were obtained between 4000 and 600 cm^−1^ using a Nicolet iS10 smart iTRBasic (Thermo Fisher Scientific, Waltham, MA, USA) model, with 64 scans and a 4 cm^−1^ resolution [8,9].

#### 2.3.2. Scanning Electron Microscopy (SEM)

The morphology and microstructure of bilayer films were observed by VP SEM Hitachi S-3400N (Hitachi, Chiyoda, Japan) using a voltage of 20.0 kV and 100.0 µA emission. Before performing SEM analysis, films were kept in a desiccator. To observe the cross-section, bilayer films were gently cut with a thin blade. Subsequently, samples were placed on appropriate stubs and spray-coated with gold using a metal evaporator (Quorum Q150R ES, East Sussex, UK) [17,18].

#### 2.3.3. Grammage, Thickness, Mechanical and Optical Properties

The grammage of the films was calculated based on the ratio between their mass and area (g/m^2^), following the ISO 536:1995. The thickness (µm) was measured according to ISO 534:2011 using a micrometer (Adamel Lhomargy Model MI 20, Veenendaal, Netherlands), with several random measurements being considered [8,9].

The mechanical properties of the films (elongation at break (%), tensile index (N·m/g) and elastic modulus (MPa)) were obtained using a tensile tester (Thwing-Albert Instrument Co., West Berlin, NJ, USA), at 23 ± 2 °C and 50 ± 5% relative humidity (RH), following the ISO 1924/1, with a single modification: the initial grip was set at 50 mm and the crosshead was set at 10 mm/min [8,9].

The color and transparency of the films were assessed using a Technidyne Color Touch 2 spectrophotometer (New Albany, IN, USA). These measurements were performed on several random positions of the films using the illuminant D65 and the observer 10°. Color coordinates L* (lightness), a* (redness; ±red-green) and b* (yellowness; ±yellow-blue) were obtained [8,9].

#### 2.3.4. Contact Angle Measurements and Surface Free Energy

The contact angles of the films were determined by the sessile drop contact angle method using a model OCAH 200 (DataPhysics Instruments, Filderstadt, Germany) that allowed image acquisition and data analysis. The surface free energy (total, dispersive and polar components) of the films was determined by measuring the contact angles with three pure liquids (deionized water, ethylene glycol and diiodomethane) [19]. The surface tension components of the reference liquids were provided by the equipment’s software [20]. Contact angle data were obtained from at least three determinations for each liquid and for each sample, and the surface free energies of the samples were obtained employing the Owens–Wendt approach [17].

#### 2.3.5. Barrier Properties

##### Water Vapor Permeability

Water vapor permeability (WVP; g/Pa·day·m) and water vapor transmission rate (WVTR; g/m^2^·day) were determined according to the standard protocol ASTM E96-00. The films, which were previously equilibrated at 23 ± 2 °C and 50 ± 5% RH, were fixed on the top of equilibrated cups containing a desiccant (15 g of anhydrous CaCl_2_, dried at 105 °C before being used). The test cups were then placed in a cabinet at 23 ± 2 °C and 50 ± 5% RH. The weight changes were monitored every 2 h over a period of 48 h. The gradient was calculated from the slope of a linear regression of the weight increase *versus* time. WVTR and WVP were calculated according to the following equations [9,15]:

WVTR = (∆*m*/∆*t*)/*A*, (1)

where ∆*m* is the weight changes of test cups (g), *A* is the test area (m^2^), and *t* is the test time (day).

WVP = WVTR/∆*p* = (WVTR/[*p*(RH_1_ − RH_2_)]) × *e*, (2)

where *p* is the vapor pressure of water at 23 °C (Pa), RH_1_ is the RH of the cabinet (50%), RH_2_ is the RH inside the cups (0%), and *e* is the thickness (m) of the films.

##### Oxygen Permeability

Oxygen transmission rate (OTR; cm^3^/m^2^·day) was measured according to ISO 15101-2:2003, using the equipment Labthink PERME^®^ OX2/230 (Jinan, China). The films with an exposed area of 5.31 cm^2^ were clamped into the diffusion cell. Pure oxygen was introduced into the outside chamber of the diffusion cell, being the permeation rate through the sample measured until reaching the steady state. Oxygen permeability (OP; cm^3^·µm/m^2^·day·kPa) was achieved by normalizing OTR with respect to the oxygen pressure (1 atm) and the thickness of the samples, using the following equation [18,21]:

OP = (OTR/∆*p*O_2_) × *e*, (3)

where *e* is the thickness of the films (µm), and ∆*p*O_2_ is the difference of oxygen partial pressure (Pa) between the two sides of the film.

These measurements were performed in duplicates for each sample at 23 ± 0.5 °C and 50 ± 2% RH.

#### 2.3.6. Antioxidant Activity

##### DPPH (2,2-diphenyl-1-picrylhydrazyl) Free Radical Scavenging Assay

For this assay, three disks of the bilayer films (6 mm of diameter) were added to 2.9 mL of a DPPH methanolic solution (0.1 mM). Then, the absorbances were measured at 517 nm every 30 min, for 5 h, against a blank of methanol. Control samples consisted in 100 µL of methanol with 2.9 mL of the DPPH methanolic solution. The antioxidant activity of the films was calculated using the following equation [8,9]:

% Inhibition = [(A_control_ − A_sample_)/A_control_] × 100, (4)

where A_control_ is the absorbance of the control, and A_sample_ is the absorbance of the sample (films).

##### β-Carotene Bleaching Test

Initially, 500 µL of a β-carotene solution (20 mg/mL in chloroform) were added to 40 µL of linoleic acid, 400 µL of Tween 40 and 1 mL of chloroform. The chloroform was then evaporated under vacuum, and 100 mL of distilled water saturated with oxygen were added to the mixture to form an emulsion. Then, 5 mL of this emulsion were pipetted into test tubes containing three disks of the bilayer films (6 mm of diameter). Finally, the tubes were shaken and placed at 50 °C in a water bath for 1 h. The absorbances of the samples were measured at 470 nm against a blank containing an emulsion without β-carotene. Control samples consisted in 5 mL of the emulsion and 300 μL of methanol. The antioxidant activity of the films was determined as the percentage of inhibition of β-carotene oxidation by the following equation [8,9]:

% Inhibition = [(A^t=1h^_samlpe_ − A^t=1h^_control_)/(A^t=0h^_control_ − A^t=1h^_control_)] × 100, (5)

where A^t=1h^ is the absorbance of the sample (films) or control at the final time of incubation, and A^t=0h^ is the absorbance of the control at the initial time of incubation.

#### 2.3.7. Antibacterial Properties

The antibacterial properties of the bilayer films against two foodborne pathogens (*Enterococcus faecalis* ATCC 29212 and *Listeria monocytogenes* LMG 16779) were evaluated by solid diffusion assay. For this test, inoculums were prepared by suspending some bacterial colonies in sterile saline solution (NaCl; 0.85%; *w*/*v*) to a cell suspension of 0.5 McFarland units (1 to 2 × 10^8^ colony-forming units/mL (CFU/mL)). Disks of the bilayer films (6 mm of diameter) were prepared under aseptic conditions, and after the inoculation of Müeller–Hinton agar (MHA) plates, they were placed over the inoculated culture medium, with the pullulan layer in contact with the agar surface. Finally, the plates were incubated at 37 °C for 18 h. After this period, the plates were visually checked for inhibition zones, being their diameters measured using a digital pachymeter. This assay was performed in three independent replicates [8,9].

### 2.4. Study of the Release Kinetics of Isopropyl Palmitate

To study the release kinetics of the major compound of licorice essential oil (isopropyl palmitate) from the developed films, the Commission Regulation on plastic materials and articles intended to contact with food EU No. 10/2011 was followed [17,22,23]. Initially, films were immersed into 15 mL of two different food simulants: 10% (*v*/*v*) ethanol that simulates aqueous food, like fresh meat products, and 50% (*v*/*v*) ethanol to simulate semi-fatty food. Then, these samples were stored during 10 days at both room temperature and 4 °C. Afterwards, aliquots (500 µL) were collected after 1 h, 2 h, 4 h, 6 h, 8 h, 1 day, 2, 3, 4, 5, 6, 7, 8, 9, and 10 days, which were subsequently filtered (0.22 µm) and stored in absence of light at 4 °C until further analysis [17,22,23].

To identify and quantify isopropyl palmitate, a simple HPLC-DAD method was developed and validated according to the guiding principles of FDA. A high-performance liquid chromatography system (HPLC) with a binary pump coupled to a diode-array detector (DAD) from Agilent Technologies (Soquimica, Lisboa, Portugal) were used. The compound was separated with an YMC-Triart PFP (5 µm, 4.6 by 150 mm) analytical column coupled to a Guard-c holder (4 by 10 mm) containing Triart PFP (5 µm, 3 by 10 mm) pre-column, all from YMC Europe GMBH (Solítica, Portugal). The HPLC-DAD worked on isocratic mode with a mobile phase composed by methanol/water (50:50), the flow rate was 1 mL/min, and the injection volume was 50 µL. The column and sampler temperatures were set at 24 and 4 °C, respectively, being the analyte detected at 230 nm. A calibration curve was prepared with several successive dilutions of isopropyl palmitate, using methanol as solvent. The samples were then transferred to the autosampler for injection into the HPLC-DAD system. The concentration of isopropyl palmitate released from the films was expressed as µg/mL, corresponding to two chromatographic analysis [24,25,26,27].

To understand the mechanism of isopropyl palmitate release, the experimental data were fitted in the Korsmeyer–Peppas model, an empirical mathematical model for drug release kinetics, which is described by the following equation [28]:

*M_t_*/*M_∞_* = *k* × *t^n^*, (6)

where *M_t_*/*M_∞_* is the fractional released compound (µg/mL), *t* is the time (s), *k* is the release constant (s^−1^), and *n* is the release exponent (dimensionless).

The release constant *k* provides information on the structural characteristics of the films formulation, whereas *n* is related to the compound release mechanism (*n* = 1, Case II transport; *n* = 0.5, Fickian diffusion; 0.5 < *n* < 1, non-Fickian diffusion) [28].

The release of isopropyl palmitate was further studied calculating its diffusion coefficient (*D*) within the polymer matrix, using the late-time approximation of Fick’s second law of diffusion described by the following equation [28]:

*M_t_*/*M*_0_ = 1 − (8/π^2^)exp[(−π^2^*D_t_*)/*e*^2^], (7)

where *M_t_* is the amount of compound (µg/mL) released at time *t* (s), *M*_0_ is the total amount of compound (µg/mL) loaded into the film, *D* is the diffusion coefficient of the compound (m^2^/s) within the polymer matrix, and *e* is the thickness of the film (m).

The experimental values were fitted in the Equations (6) and (7) with the GRG nonlinear solving method, using the Solver add-in from Microsoft Excel.

### 2.5. Statistical Analysis

The results were expressed as mean ± standard deviation (SD). The data were analyzed using the statistical program IBM SPSS Statistics 25. Significant differences among means were analyzed by Student’s *T*-test (assuming the normal distribution of the continuous variables). A level of *p*-value < 0.05 was considered significant.

## 3. Results and Discussion

### 3.1. FTIR Analysis

**Figure 1:**
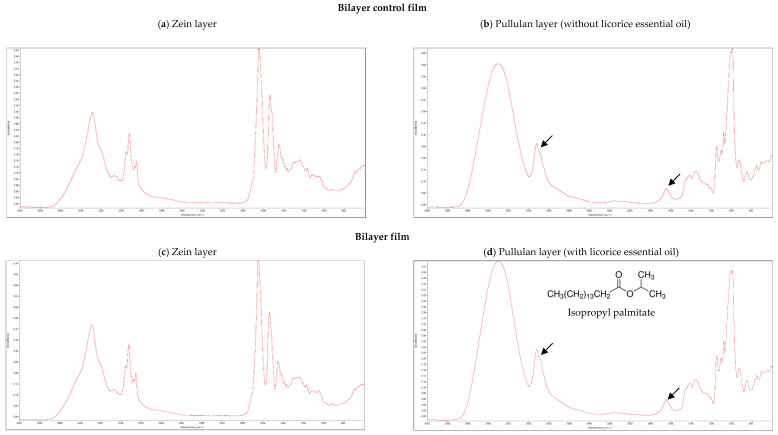
Fourier-Transform Infrared Spectroscopy (FTIR) spectra of the bilayer control film (**a**) zein layer, (**b**) pullulan layer; and bilayer film with licorice essential oil (**c**) zein layer, (**d**) pullulan layer.3.2. Microstructure of Bilayer Films.

The incorporation of licorice essential oil as active agent in bilayer films was analyzed by FTIR. Isopropyl palmitate (chemical structure shown in Figure 1d), is an hexadecenoic acid isopropyl ester, possessing an aliphatic chain with several methyl groups (CH_2_), and it is an ester, presenting a carbonyl group (C=O). The methyl groups of aliphatic chains have their characteristic infrared band at 2960 cm^−1^, while the infrared band of carbonyl groups of esters appear at 1740 cm^−1^ [8,9].

Figure 1 shows the FTIR spectra of both layers of the films with and without licorice essential oil incorporated. Zein layers presented similar FTIR spectra in both types of films (Figure 1a,c), also being analogous to the FTIR spectra of zein-based films previously developed by our research group [9]. These results indicate that licorice essential oil, when incorporated in the pullulan layer, did not migrate between the layers of the films. This result is significant since the main objective is the migration of the essential oil from the internal layer of pullulan to the packaged food and not to the external layer of zein. Observing the FTIR spectra of pullulan layers (Figure 1b,d), the characteristic infrared bands of methyl and carbonyl groups of isopropyl palmitate, which are expected to be present only when licorice essential oil is incorporated in pullulan, were found in both type of films (marked with arrows). This observation may be explained by the presence of Tween 40 in both types of films. This surfactant was used as emulsifier, since it is often used in some pharmaceuticals and food preservation, being also employed in cosmetics to solubilize essential oils into water-based products [29]. Tween 40 is an ester presenting also a long aliphatic side chain. Therefore, the band of methyl groups of the aliphatic chain found on isopropyl palmitate and the band of its carbonyl group are overlapped with the same infrared bands of Tween 40, which was added in both types of films, making it impossible to distinguish the FTIR spectra of the pullulan layers (with and without licorice essential oil incorporated), contrariwise to what was observed in previous works where Tween 40 was not used [8,9].

### 3.2. Microstructure of Bilayer Films

**Figure 2:**
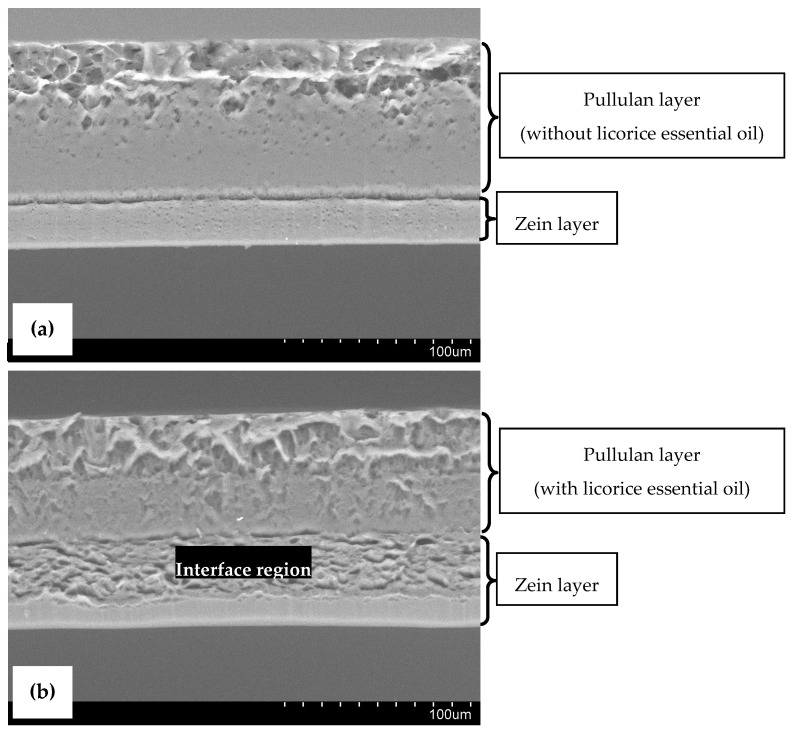
Scanning Electron Microscopy (SEM) images of cross-sections of bilayer control film (**a**), and bilayer film incorporating licorice essential oil (**b**). (Magnification: 500×).

SEM micrographs of cross-sections of the designed bilayer films are presented in Figure 2, where the two layers can be distinguished. The bilayer control film (Figure 2a) exhibited an overall continuous and compact microstructure, confirming the adhesion of the two layers. The pullulan layer shows the presence of some microdroplets entrapped in the film matrix, a result of the homogenization procedure. Otherwise, the bilayer film with licorice essential oil (Figure 2b) seems to be smoother and less compacted. The presence of microdroplets is even more evident in this film, indicating the proper incorporation of licorice essential oil in the biopolymeric matrix. Moreover, the zein layer appears to be different, probably because of chemical interactions that occur between the essential oil and zein molecules via hydrogen bonds in the interface region (highlighted in Figure 2b) of both layers of the film, presenting high essential oil concentration, as it was reported earlier by other authors [5]. This interface region between the two layers of the film with licorice essential oil suggests the potential migration of the essential oil from the pullulan layer (more hydrophilic) to the zein layer (more hydrophobic), which was not verified by FTIR analysis, as mentioned above. To avoid the formation of this interface region, the bilayer films incorporating licorice essential oil may be subjected to calendaring molding with a combination of temperature and pressure, which may negatively impact their general physical properties [21].

### 3.3. Grammage, Thickness, Mechanical and Optical Properties

The grammage and thickness of bilayer films incorporating licorice essential oil were significantly higher (*p*-value < 0.05) than those of bilayer control films (Table 1). These results may be explained by the higher total solids content when the essential oil is present. Moreover, the mixture of a lipophilic substance, like the essential oil, in the aqueous pullulan film-forming solution may result in the entrapment of air microdroplets in the biopolymeric matrix, thereby decreasing the compactness of the pullulan film, as other authors have also reported [30]. In this work, even though the film-forming solution was degasified under vacuum before the casting procedure, the addition of Tween 40 and xanthan gum increased the viscosity of the solution, blocking the release of entrapped air microbubbles.

The mechanical properties of films are important because they are related to the end-use characteristics of these materials [30]. Elongation at break and elastic modulus of the developed bilayer films were not significantly influenced (*p*-value > 0.05) by the addition of licorice essential oil (Table 1), contrariwise to what is usually observed as most of the published data reported a decrease in elongation after essential oils incorporation [30]. In the bilayer films this effect was not observed, most likely due to the overall reinforcement of the mechanical properties achieved by combining two biopolymeric layers with distinct characteristics. Concerning the tensile index, a significant decrease (*p*-value < 0.05) was observed when licorice essential oil was incorporated (Table 1), which indicates that the essential oil decreases the strength of the films.

The optical properties of the bilayer films were significantly affected (*p*-value < 0.05) when licorice essential oil was incorporated (Table 1). Color coordinates (L*, a*, b*), particularly the yellowness (b*) were higher in bilayer films with licorice essential oil, which is due to the yellow-brownish color of the essential oil. Additionally, the transparency of the films decreased from 92.10% to 88.05% with the incorporation of the essential oil. This result may be explained by the formation of an emulsion when the essential oil is incorporated, favoring the increase of films opacity. Though, 88.05% of transparency is a good result when compared to values of transparency near to 3% obtained for gelatin-chitosan-pectin films incorporated with rosemary essential oil [31].

### 3.4. Contact Angles and Surface Free Energy

The data obtained through the contact angle measurements provides information on the hydrophobicity/hydrophilicity of the bilayer films surface. The total surface free energy of a solid can be expressed as the sum of the dispersive and polar components. The Owens–Wendt equation applies the data of the polar and non-polar liquids, with dispersive and polar components, total surface free energy and contact angle [32].

Observing the results of the contact angles obtained for the three pure liquids, it was verified that no significant differences (*p*-value > 0.05) within the zein and pullulan layers were observed (Table 2). The incorporation of licorice essential oil on pullulan did not affected its hydrophilicity. Usually, a surface is classified as hydrophobic when its water contact angle is higher than 90° [33]. Zein layers had a contact angle of approximately 70°, indicating its hydrophobic potential, as expected (Table 2). Regarding pullulan layers, their water contact angles were close to 43°, indicating their hydrophilic character (Table 2).

The absence of significant differences (*p*-value > 0.05) amongst the contact angles was also reflected on the surface free energy (total, dispersive and polar components) (Table 2). The polar component of pullulan layers (≈36 mN/m) corroborates their hydrophilic character, which is in agreement with the hygroscopic behavior presented by pullulan. This is useful to facilitate the controlled release of isopropyl palmitate to the packaged food, whereas the hydrophobic character of zein layer provide better barrier properties to the films, improving the overall shelf-life of the packaged food. Moreover, higher total surface free energy, as obtained for pullulan layers, may help in the control of the wettability characteristics of the bilayer films [34].

### 3.5. Barrier Properties

Functional edible films must be designed to meet several requirements. In addition to proper mechanical properties and good appearance (linked to optical properties), they should present appropriate barrier properties, namely to water vapor and oxygen [35], which were also evaluated.

Table 3 shows the water vapor and oxygen permeabilities of the developed bilayer films. The values of water vapor permeability (WVTR and WVP) did not change significantly (*p*-value > 0.05) when licorice essential oil was incorporated (Table 3). The bilayer films presented better barrier properties, concerning the water vapor permeability, than those developed earlier composed by an unique layer of zein [9]. The WVTR of bilayer films are close do 16 g/m^2^·day and of the zein-based films previously reported are around 30 g/m^2^·day [9], indicating that bilayer films allow less permeation of water vapor through them. This is a relevant improvement of the barrier properties that will contribute to increased preservation of the packaged food. Other authors observed a decrease in WVP as a result of essential oils incorporation, suggesting that the diffusivity of water molecules decreased because of the hydrophobic nature of the essential oils [36].

The incorporation of licorice essential oil significantly decreased (*p*-value < 0.05) the oxygen permeability (OTR and OP) of bilayer films (Table 3), improving their barrier properties. The chemical affinity of permeant and film greatly affect the permeability values [35]. In this sense, the lowest oxygen permeability observed for bilayer films incorporated with the essential oil can be related with some chemical repulsions that may occur between oxygen and licorice essential oil. Furthermore, the interactions between the essential oil components, such as isopropyl palmitate, with the hydroxyl groups in the biopolymeric matrix of the films, could result in a more ordered and tightly crosslinked structure, and consequently a lower oxygen permeability [36].

The cast-supporting surface together with the incorporation of the essential oil can lead to the formation of pinholes in the film structure, which will facilitate the permeation of water vapor and oxygen. The obtained results for barrier properties of the bilayer films developed in this work do not suggest the pinholes formation when licorice essential was incorporated in the films, particularly in the interface region.

### 3.6. Antioxidant and Antibacterial Activities

**Figure 3:**
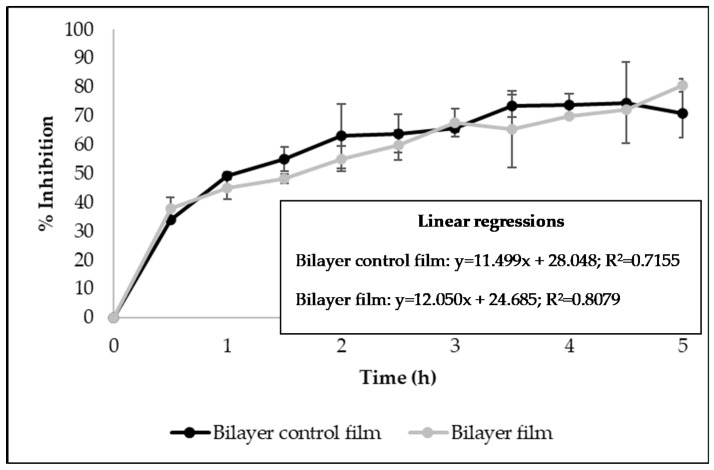
Antioxidant activity of bilayer films measured by DPPH free radical scavenging assay (Results expressed as mean ± SD).

The antioxidant activity of bilayer films developed in this work was studied using the DPPH free radical scavenging assay and the β-carotene bleaching test. Figure 3 shows the results of the DPPH assay, presented as Percentage Inhibition measured over time (5 h). It was verified that control bilayer films and the ones incorporating licorice essential oil presented similar radical scavenging activity (Figure 3), indicating that the addition of the essential oil did not increase that activity. Due to its chemical structure, zein has great potential for use in food packaging because of its intrinsic antioxidant nature, allowing zein-based films to have antioxidant activity without adding any antioxidant during processing [37]. It was also verified by the linear regressions that the ability of the films to scavenge the DPPH free radicals occurs in a time-dependent manner (R^2^ > 0.7) (Figure 3).

Bearing in mind the potential application of the bilayer films to package foods with high fat content, their capacity to inhibit the lipid peroxidation was evaluated by β-carotene bleaching test (Table 4). Despite control film presented 39.86%, the incorporation of licorice essential oil significantly increased (*p*-value < 0.05) this activity to 91.89% (Table 4).

The overall antioxidant activity of bilayer films with licorice essential oil incorporated, related with the ability to scavenge free radicals and to inhibit the lipid peroxidation, indicates their potential application to package foods presenting fat in its composition, avoiding the rancidity.

*E. faecalis* and *L. monocytogenes* are microorganisms able to adhere to several food-use approved materials including metals, rubbers, and polymers [38], causing infections in humans when ingesting contaminated foods of animal origin [39]. Licorice essential oil shown to be effective in inhibiting both the growth and the biofilm formation of these two foodborne pathogens [8].

The antibacterial activity of bilayer films was evaluated against *E. faecalis* and *L. monocytogenes* (Table 4). It was verified the bacterial cells retraction at the contact area of bilayer films with licorice essential oil together with a clear inhibition zone around the disks of this type of films (Table 4). These results suggest the possible application of the developed bilayer films incorporating licorice essential oil to package foods susceptible to contamination by *E. faecalis* and *L. monocytogenes*.

### 3.7. Release Kinetics of Isopropyl Palmitate

When developing bioactive films as future food packaging materials, it is important to understand in what conditions they would be able to be applied, namely, the type of food and the storage conditions. Thus, in this work, the ability of the developed bilayer films to control-release the active agent over time was evaluated, studying the release kinetics of isopropyl palmitate (the major compound of licorice essential oil) under different conditions. This study was performed over 10 days, using two food simulants to simulate aqueous food (10% ethanol) and semi-fatty food (50% ethanol), under two temperatures corresponding to those where most of food products are frequently stored (room temperature and 4 °C).

**Figure 4:**
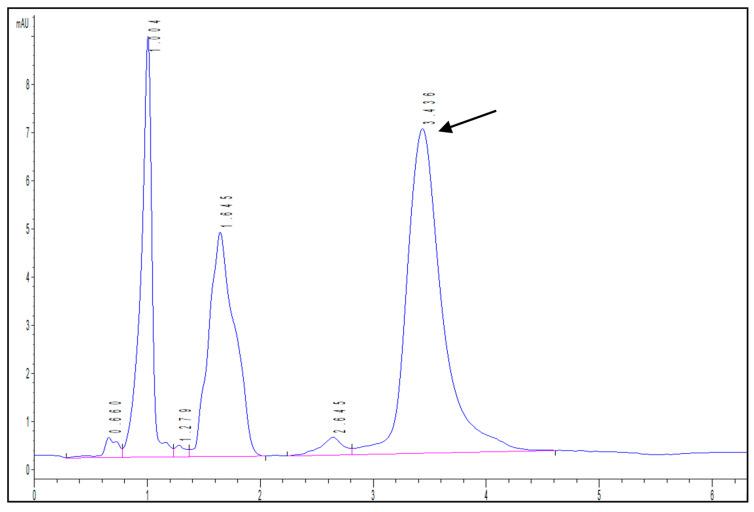
Example of HPLC-DAD chromatogram showing the peak corresponding to isopropyl palmitate (retention time = 3.436 min).

Firstly, a HPLC-DAD method was developed to identify and quantify isopropyl palmitate. Figure 4 shows an example of a chromatogram obtained during the assays, highlighting the peak corresponding to the compound at 3.436 min.

**Figure 5:**
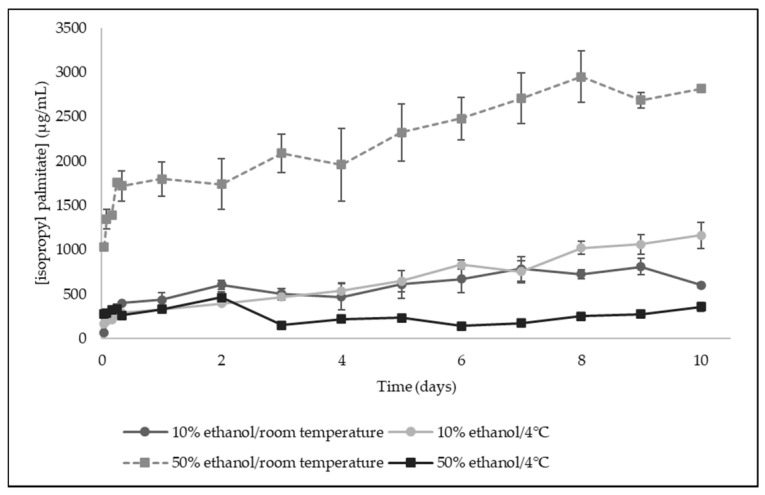
Release kinetics of isopropyl palmitate from bilayer films to food simulants (10% ethanol and 50% ethanol) at room temperature and 4 °C (Results expressed as mean ± SD).

The release kinetics profile of isopropyl palmitate from bilayer films incorporating licorice essential oil is shown in Figure 5. It was observed that in 50% ethanol at room temperature, the release was more effective, while in the remaining conditions seems to be very similar. These results suggest that the bilayer films will be more efficient if applied to package semi-fatty food. Isopropyl palmitate is a very hydrophobic compound, presenting an octanol/water partition coefficient (logP) of 8.16, which is difficult to release in 10% ethanol, a food simulant mainly composed by water, where the compound is immiscible.

Licorice essential oil was incorporated in the pullulan internal layer of the films. Polysaccharides, like pullulan, will undergo hydrolysis at higher temperatures, which together with its dissolution in the food simulant, due to solvent penetration effect, swelling, and polymer chain disentanglement and relaxation, allows the compound transport from bilayer films by diffusion and/or dissolution [28]. This may explain the release profile of isopropyl palmitate in 50% ethanol at room temperature, reflecting the influence not only of the food simulant used, but also the impact of the temperature, a factor of utmost importance when studying the release kinetics of compounds from biopolymeric-based films [28].

The release profile of isopropyl palmitate from the developed films was subjected to release kinetic modelling using the Korsmeyer–Peppas model. The experimental data obtained in this study presented better linearity (higher linearity coefficient - R^2^-values) when *n* = 0.5, corresponding to a Fickian diffusion. The release constant of isopropyl palmitate was then determined for all the experimental conditions (Table 5). The higher release constant was obtained for 50% ethanol at room temperature (*k* = 45.40 × 10^−5^ s^−1^; error = 1.00 × 10^−14^), indicating that the release of isopropyl palmitate will be facilitated by higher temperatures like room temperature.

Since the release kinetics of isopropyl palmitate follows a Fickian diffusion, it was further subjected to mathematical modelling, using the late-time approximation of Fick’s second law of diffusion, assuming that the dimension and physical properties of the bilayer films matrix do not change during the release process, i.e., there is no degradation or mass loss of the bulk materials [28]. The application of this diffusion-controlled model allows the determination of isopropyl palmitate diffusion coefficient for each experimental condition (Table 6).

The results showed that the highest diffusion coefficient was obtained for 50% ethanol at room temperature (*D* = 1.004 × 10^−9^ m/s^2^; error = 8.089 × 10^−5^), corroborating the experimental results, indicating the efficient release of isopropyl palmitate on those conditions (room temperature, 50% ethanol). Other authors reported that the release of bioactive agents from films is kinetically inhibited under cold temperatures and favored at warmer temperatures [40].

## 4. Conclusions

In this work, a completely biodegradable and biocompatible bilayer film (zein/pullulan) incorporating licorice essential oil as active agent was successfully designed and characterized. The developed bilayer films showed overall good mechanical, optical and barrier properties. Moreover, these films were able to inhibit lipid peroxidation and the growth of two known foodborne pathogens, indicating their potential application in the preservation of food quality while replacing the use of petroleum-based plastics. The release kinetics of isopropyl palmitate suggests that the bilayer films will be more efficient when used to package foods with high fat content stored at room temperature. Future research should focus on scale-up production of these new edible and bioactive food packaging materials.

## Figures and Tables

**Table 1:** Grammage, thickness, mechanical and optical properties of bilayer films.

Properties		Bilayer Control Films	Bilayer Films (with Licorice Essential Oil)	*p*-Value
Grammage (g/m^2^)		134.38 ± 2.13	149.16 ± 1.74	0.001 *
Thickness (µm)		154.16 ± 6.63	168.37 ± 5.42	0.047 *
Mechanical properties	Elongation at break (%)	3.00 ± 0.01	2.39 ± 0.36	0.099
	Tensile index (N·m/g)	25.33 ± 0.93	20.33 ± 1.67	0.018 *
	Elastic modulus (MPa)	1196.92 ± 149.41	1119.66 ± 62.99	0.476
Optical properties	L* (lightness)	31.87 ± 0.37	38.45 ± 0.44	<0.001 *
	a* (redness)	−0.61 ± 0.03	−0.17 ± 0.03	<0.001 *
	b* (yellowness)	17.68 ± 0.88	22.95 ± 1.04	0.003 *
	Transparency (%)	92.10 ± 0.21	88.05 ± 0.17	<0.001 *

Results expressed as mean ± SD; * indicates a significant result (*p*-value < 0.05).

**Table 2:** Contact angles and surface free energy (ɤ) of bilayer films.

Properties	Bilayer Control Films		Bilayer Films		*p*-Values
	Zein Layer ^a^	Pullulan Layer (without Licorice Essential Oil) ^b^	Zein Layer ^c^	Pullulan Layer (with Licorice Essential Oil) ^d^	
Water contact angle (°)	68.79 ± 1.25	44.22 ± 1.11	71.73 ± 2.29	43.71 ± 1.41	0.143 ^ac^ 0.650 ^bd^
Ethylene glycol contact angle (°)	58.64 ± 1.86	61.97 ± 2.78	61.23 ± 0.61	61.76 ± 2.49	0.127 ^ac^ 0.927 ^bd^
Diiodomethane contact angle (°)	36.20 ± 1.70	37.20 ± 1.29	36.35 ± 1.94	38.22 ± 2.07	0.925 ^ac^ 0.516 ^bd^
Total surface free energy, ɤ^T^ (mN/m)	39.65 ± 1.98	52.89 ± 2.64	38.05 ± 1.90	53.14 ± 2.66	0.370 ^ac^ 0.914 ^bd^
Dispersive component, ɤ^D^ (mN/m)	28.41 ± 1.42	16.74 ± 0.84	28.17 ± 1.41	16.60 ± 0.83	0.846 ^ac^ 0.847 ^bd^
Polar component, ɤ^P^ (mN/m)	11.24 ± 0.56	36.15 ± 1.81	9.88 ± 0.66	36.54 ± 1.83	0.054 ^ac^ 0.806 ^bd^

Results expressed as mean ± SD; upper letters were used to identify the pairs of samples under statistical comparison.

**Table 3:** Barrier properties of bilayer films.

Permeability		Bilayer Control Films	Bilayer Films (with Licorice Essential Oil)	*p*-Value
Water vapor	WVTR (g/m^2^·day)	16.24 ± 0.68	15.53 ± 0.34	0.206
	WVP (g/Pa·day·m) (×10^−6^)	1.89 ± 0.08	1.98 ± 0.04	0.182
Oxygen	OTR (cm^3^/m^2^·day)	1845.15 ± 164.25	1161.33 ± 6.69	0.019 *
	OP (cm^3^·µm/m^2^·day·kPa)	3041.00 ± 246.89	2143.00 ± 168.29	0.009 *

Results expressed as mean ± SD; * indicates a significant result (*p*-value < 0.05).

**Table 4:** Antioxidant activity (β-carotene bleaching test) and antibacterial properties of bilayer films.

Properties		Bilayer Control Films	Bilayer Films (with Licorice Essential Oil)	*p*-Value
β-carotene bleaching test	% Inhibition	39.86 ± 0.83	91.89 ± 1.07	<0.001 *
Inhibition zones (mm)	*E. faecalis* ATCC 29212	6.00 ± 0.00 (−)	7.02 ± 0.56 (+)	0.087
	*L. monocytogenes* LMG 16779	6.00 ± 0.00 (−)	6.84 ± 0.48 (+)	0.094

Results expressed as mean ± SD; (−) bacterial growth on the top of the films; (+) bacterial cells retraction at the contact area and clear inhibition zone; * indicates a significant result (*p*-value < 0.05).

**Table 5:** Release constant of isopropyl palmitate determined using Korsmeyer–Peppas model (*n* = 0.5, Fickian diffusion).

Conditions		Release Constant, *k* (s^−1^) (×10^−5^)	Error (×10^−14^)
10% ethanol	room temperature	9.43	1.28
	4 °C	6.29	1.00
50% ethanol	room temperature	45.40	1.00
	4 °C	9.00	1.28

**Table 6:** Diffusion coefficient of isopropyl palmitate determined using the late-time approximation of Fick’s second law of diffusion.

Conditions		Diffusion Coefficient, *D* (m^2^/s) (×10^−9^)	Error (×10^−5^)
10% ethanol	room temperature	0.363	8.472
	4 °C	0.250	5.706
50% ethanol	room temperature	1.004	8.089
	4 °C	0.142	1.642
